# Corruption and inequality of wealth amongst the very rich

**DOI:** 10.1007/s11135-015-0202-4

**Published:** 2015-04-21

**Authors:** Philip Hans Franses, Bert de Groot

**Affiliations:** Erasmus School of Economics, Econometric Institute, Burgemeester Oudlaan 50, 3062 PA Rotterdam, The Netherlands

**Keywords:** Corruption, Inequality, Wealth, D73, D31

## Abstract

Corruption may lead to tax evasion and unbalanced favors and this may lead to extraordinary wealth amongst a few. We study for 13 countries 6 years of Forbes rankings data and we examine whether corruption leads to more inequality amongst the wealthiest. When we correct in our panel model for current and one-year lagged competitiveness and GDP growth rates, we find no such effect. In fact, we find that more competitiveness decreases inequality amongst the wealthiest.

## Introduction and motivation

Corruption is an important topic to study as it has been shown to affect economic growth and inequality amongst individuals, see Shleifer and Vishny (1993) and Mauro (1995, 2004) and Husted (1999) for classic general studies and Ravallion and Chen (1997), Mo (2001), Jain (2001), Wilhelm (2002), Gyimah-Brempong (2002) and Gupta et al. (2002) for more specific accounts. A general finding is that higher levels of corruption lead to more inequality and more poverty, meaning lower incomes at the lower end. In the present paper we aim to add to the knowledge base by looking only at the wealth levels at the top end and we examine if inequality amongst the wealthiest is associated with corruption.

Income differences at the top end can be rather large, and they are worthwhile to study. Some companies give enormous bonuses to their board members, while others follow more restrictive guidelines. Some managers allot large amounts of stocks and options to themselves. One could argue that a business community in a country should strive for some degree of equality, also from an ethical viewpoint but also from the viewpoint of the ties that exist between various companies. Sharply differing remuneration levels will out price certain leaders, while newer firms will never be able to afford these board members. This raises ethical questions on the link between corruption and income inequality at the top end. Recent research on the ethical issues in business includes Halter and Arruda (2009), Aguilera and Vadera (2008), Bishara and Schipani (2009) and Hess (2009), and Pelletier and Bligh (2006), (2008) and Méon and Weill (2010), among others.

Gupta et al. (2002) and Jain (2001) argue that corruption can lead to tax evasion or otherwise disproportionate favors to only a few. This would imply that some individuals can become exceptionally rich. Hence, not only could corruption lead to more poverty at the bottom end of the income spectrum, it could also lead to exceptional wealth for only a few. A casual glance at the Forbes lists for countries like Indonesia, China and Thailand could suggest this correlation indeed. Moreover, Neumayer (2004) and Torgler and Piatti (2009, 2013) study the number of billionaires within countries and correlates these numbers with various variables like corruption, GDP and population size. It is found that corruption makes the number of superrich to increase. In this paper we do not focus on the number of superrich, but merely we examine the wealth inequality amongst those very wealthy individuals.

In sum, we examine the same issue from a different angle. We study if inequality amongst the very wealthiest also increases with corruption, that is, are there amongst the richest only a few with perhaps excessive fortune? Indeed, it is usually found that corruption leads to more inequality, but does this also hold for the very rich?

Following the literature, we include in our empirical econometric model also measures of competition and GDP, as these may also influence inequality. And, to overcome endogeneity issues, we also consider a panel model where we only include the one-year lagged data on the explanatory variables. Various versions of our panel model all lead to one and the same conclusion, and that is that is not corruption that drives inequality amongst the wealthiest but it is competitiveness. The least competitive is a country the larger is the difference in wealth amongst the superrich. To check for potential confounding effects, at the same time we show that the measures on competitiveness and corruption do not correlate much.

In Sect. 2 we outline the construction of the database, and in Sect. 3 we present the estimation results. Section 4 concludes.

## Data

We start with the data on inequality amongst the superrich. For this, we consult the Forbes lists for 13 countries.^1^ These countries are Australia (where the list contains 40 entries), China (400), Hong Kong (40), India (100), Indonesia (40), Japan (40), Korea (40), Malaysia (40), the Philippines (40), Singapore (40), Taiwan (40), Thailand (40) en the United States of America (500).

**Fig. 1:**
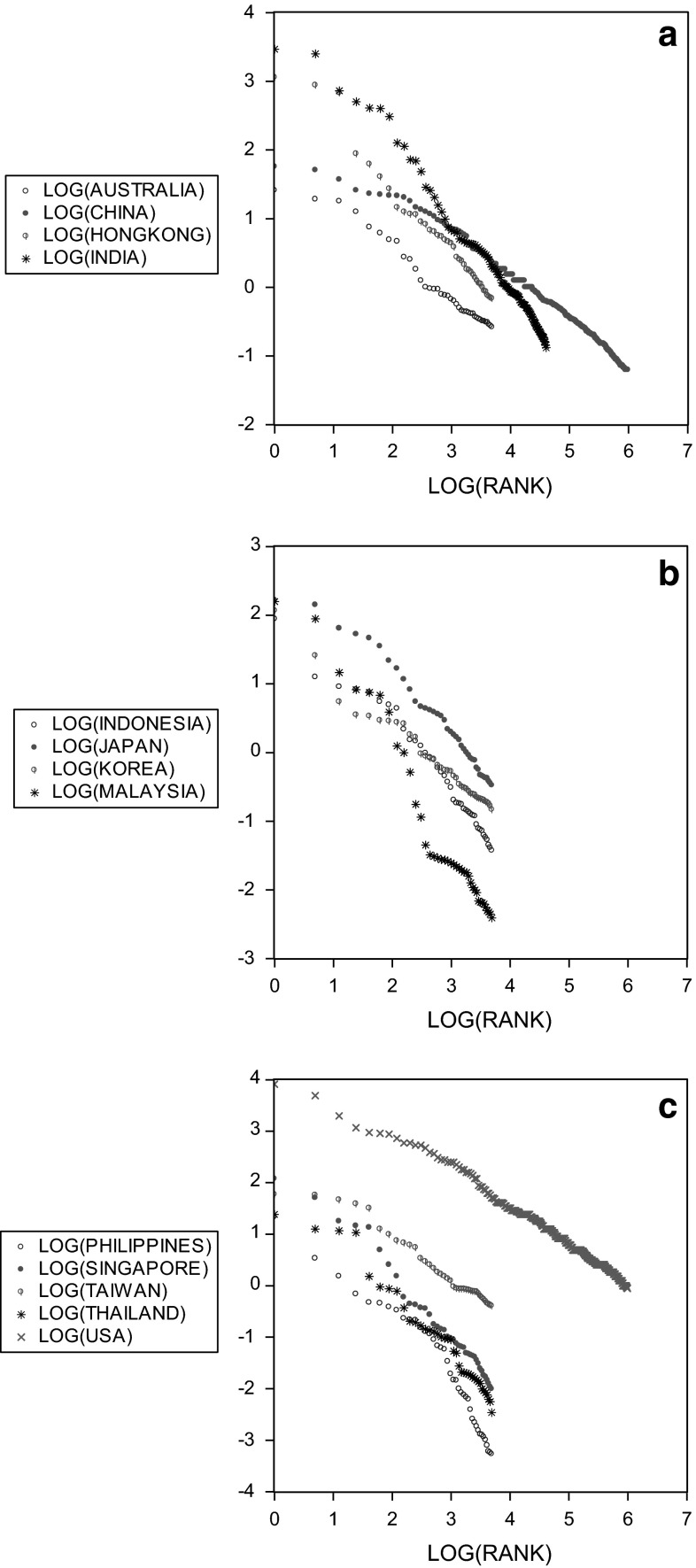
**a** Scatter of the log of wealth (in billions of USD) against the log of the rank (2009 data). **b** Scatter of the log of wealth (in billions of USD) against the log of the rank (2009 data). **c** Scatter of the log of wealth (in billions of USD) against the log of the rank (2009 data)

Klass et al. (2006) have shown that the Forbes ranking for the USA obeys a power law. Using an alternative ranking for the superrich in the Netherlands, Franses en Vermeer (2012) document similar results, and that is that the differences in wealth of those at ranks, say, 2 and 3, is similar to the differences between ranks 3 and 4. This can be visualized by plotting the natural logarithm of wealth against the natural logarithm of the associated rank. Figure 1a–c show these linear links for the 2009 Forbes rankings, as an illustration. For other years, similar graphs appear. The slopes of these lines (when approximated using a linear regression model) are called alpha. Clearly, the more negative is alpha the larger are the differences in wealth of the wealthiest. In Table 3 in the Appendix we present the estimates of alpha for the 13 countries for 2006–2011, when available.

In the Data Appendix, we also present the data on the explanatory variables that we use in our panel model below. The source of our corruption data is Transparency International.^2^ The numbers in our table are 10 minus the scores, where now the corruption figures are such that higher values mean more corruption. In the literature on income inequality, there are several explanatory factors considered, and the commonly considered variables are a measure of competitiveness and GDP growth. The data for competitiveness are drawn from the World Economic Forum,^3^ see Appendix Table 5. For GDP growth, we consult the database of the World Bank.^4^ In Appendix Table 6 contains the data on this last variable.

A priori, we would expect that more corruption would lead to more inequality, also amongst the wealthiest. At the same time, a higher level of competitiveness means that there are more companies which survive and succeed, and this would lead to more equality, also amongst the superrich. Finally, higher economic growth comes to the benefit of many individuals, and, as indicated in the relevant literature, there we would expect a decreasing effect on inequality, also for the superrich.

In Table 1 we present the correlations across the explanatory variables for each of the countries. It is interesting to see that the correlations are usually quite small. Also, there even seems to be no common sign of the correlations as positive and negative correlations appear about equally frequently.

**Table 1:** Correlations across explanatory variables

Country	Corruption-competitiveness	Corruption-GDP growth	Competitiveness-GDP growth
Australia	0.280	0.579	0.557
China	−0.860	0.676	−0.696
Hong Kong	−0.257	−0.650	0.761
India	−0.038	−0.130	0.294
Indonesia	−0.698	−0.016	0.449
Japan	0.125	−0.018	0.284
Korea	0.346	0.637	0.051
Malaysia	−0.569	−0.246	0.493
Philippines	−0.132	−0.066	0.119
Singapore	−0.033	−0.685	−0.317
Taiwan	−0.460	−0.366	0.198
Thailand	0.700	−0.025	0.137
USA	−0.548	0.835	−0.349

## Empirical analysis

To link the alpha measure for inequality with the explanatory variables, we consider versions of the following panel data model, that is

$$ alpha_{i,t} = \mu_{i} + \rho\,  alpha_{i,t - 1} + \beta_{1} \,corruption_{i,t} + \beta_{2} \,corruption_{i,t - 1} + \gamma_{1}\, competitiveness_{i,t} + \gamma_{2} \,competitiveness_{i,t - 1} + \delta_{1} GDPgrowth_{i,t} + \delta_{2} GDPgrowth_{i,t - 1} + \varepsilon_{i,t} $$

We have to set most of the parameters (except for the country-specific intercept) as equal across the countries, in order to gain degrees of freedom. As for some countries alpha estimates are missing, our model is an unbalanced panel model.

Some of the most relevant least-squares-based estimation results are presented in Table 2. Other versions of the model (no lags, and no one-year lagged alpha) give qualitatively similar outcomes. Clearly, the only variable that is relevant to explain inequality amongst the wealthiest is the measure of competitiveness. More competition leads to less inequality. This is a conclusion that has been drawn before and which is reiterated here when looking only at the wealthiest individuals in 13 countries. Corruption seems not to have much of an effect. The sign is correct though, implying that more corruption associates with more inequality, but the estimate is not significant. Perhaps when more data become available in the future the potential relevance of this variable can be explored further.

**Table 2:** Various parameter estimates (with standard errors) obtained using OLS to the unbalanced panel data model

Variables	Full model	Only lags
Alpha, lagged	0.063 (0.150)	0.128 (0.153)
Corruption	−0.019 (0.071)	
Corruption, lagged	−0.128 (0.077)	−0.112 (0.073)
Competitiveness	***0.375*** (***0.160***)	
Competitiveness, lagged	0.098 (0.124)	***0.250*** (*0.106*)
GDP growth/100	0.005 (0.344)	
GDP growth/100, lagged	0.028 (0.363)	0.343 (0.329)

5 % significant parameters are in bold and italic

## Conclusion

We have demonstrated, and in contrast to income levels at the bottom end, that corruption does not seem to impact inequality of wealth amongst the superrich. In fact, when such inequalities could be reduced it could be done by increasing competitiveness. Reducing monopolies and cartels seems a better strategy to trim down the wealth of the wealthiest.

The limitations of our study are given by the data that we use. The Forbes rankings involve a considerable amount of judgment, and measurement errors can occur. As we do not use the actual data but the estimated slopes in a regression model, we hope that any measurement errors do not have too large a consequence. Similar arguments about judgment can be made for the corruption data, and there we have to rely on the quality and experience of the data compilers. A final limitation is of course that only have thirteen countries with data, and this can be considered a small sample. Unfortunately, we are not familiar with other Forbes rankings, so this limitation is beyond our efforts.

## Appendix

See Appendix Tables 3, 4, 5, 6.

**Table 3:** The data on alpha (estimated using linear regressions, rounded at three digits)

Country	Year					
	2006	2007	2008	2009	2010	2011
Australia	−0.760	−0.756	−0.759	−0.707	−0.651	−0.813
China	−0.659	−0.790	−0.580	−0.631	−0.599	−0.573
Hong Kong			−1.025	−0.987	−0.947	−0.948
India	−1.027	−1.139	−1.063	−1.053	−1.012	−1.001
Indonesia	−1.093	−1.399	−1.065	−0.935	−0.878	−0.857
Japan	−0.763	−0.709	−0.789	−0.843	−0.860	
Korea		−0.567	−0.537	−0.618	−0.714	−0.778
Malaysia	−1.430	−1.339	−1.411	−1.421	−1.468	−1.395
Philippines	−1.363	−1.326	−1.244	−1.282	−1.266	−1.259
Singapore	−1.278	−1.119	−1.167	−1.194	−1.173	−1.116
Taiwan			−0.775	−0.687	−0.726	−0.663
Thailand	−1.145	−0.962	−1.114	−1.125	−1.133	−1.125
USA	−0.723	−0.703	−0.720	−0.736	−0.742	−0.764

**Table 4:** The data of corruption

Country	Year					
	2006	2007	2008	2009	2010	2011
Australia	1.3	1.4	1.3	1.3	1.3	1.2
China	6.7	6.5	6.4	6.4	6.5	6.4
Hong Kong	1.7	1.7	1.9	1.8	1.6	1.6
India	6.7	6.5	6.6	6.6	6.7	6.9
Indonesia	7.6	7.7	7.4	7.2	7.2	7.0
Japan	2.4	2.5	2.7	2.3	2.2	2.0
Korea	4.9	4.9	4.4	4.5	4.6	4.6
Malaysia	5.0	4.9	4.9	5.5	5.6	5.7
Philippines	7.5	7.5	7.7	7.6	7.6	7.4
Singapore	0.6	0.7	0.8	0.8	0.7	0.8
Taiwan	4.1	4.3	4.3	4.4	4.2	3.9
Thailand	6.4	6.7	6.5	6.6	6.5	6.4
USA	2.7	2.8	2.7	2.5	2.9	2.9

**Table 5:** The data of competition

Country	Year					
	2006	2007	2008	2009	2010	2011
Australia	5.29	5.17	5.20	5.15	5.11	5.11
China	4.24	4.57	4.70	4.74	4.84	4.90
Hong Kong	5.46	5.37	5.33	5.22	5.30	5.36
India	4.44	4.33	4.33	4.30	4.33	4.30
Indonesia	4.26	4.24	4.25	4.26	4.43	4.38
Japan	5.60	5.43	5.38	5.37	5.37	5.40
Korea	5.13	5.40	5.28	5.00	4.93	5.02
Malaysia	5.11	5.10	5.04	4.87	4.88	5.08
Philippines	4.00	3.99	4.09	3.90	3.96	4.08
Singapore	5.63	5.45	5.53	5.55	5.48	5.63
Taiwan	5.41	5.25	5.22	5.20	5.21	5.26
Thailand	4.58	4.70	4.60	4.56	4.51	4.52
USA	5.61	5.67	5.74	5.59	5.43	5.43

**Table 6:** The data of GDP growth

Country	Year					
	2006	2007	2008	2009	2010	2011
Australia	3.1	3.6	3.8	1.4	2.3	1.8
China	12.7	14.2	9.6	9.2	10.4	9.3
Hong Kong	7.0	6.4	2.3	−2.7	7.0	5.2
India	9.3	9.8	3.9	8.2	9.6	6.9
Indonesia	5.5	6.3	6.0	4.6	6.2	6.5
Japan	1.7	2.2	−1.0	−5.5	4.4	−0.7
Korea	5.2	5.1	2.3	0.3	6.3	3.6
Malaysia	5.8	6.5	4.8	−1.6	7.2	5.1
Philippines	5.2	6.6	4.2	1.1	7.6	3.7
Singapore	8.8	8.9	1.7	−1.0	14.8	4.9
Taiwan	5.4	6.0	0.7	−1.8	10.7	4.0
Thailand	5.1	5.0	2.5	−2.3	7.8	0.1
USA	2.7	1.9	−0.4	−3.5	3.0	1.7

## Footnotes

1 http://www.forbes.com/lists/.

2 http://www.transparency.org/.

3 http://www.weforum.org/.

4 http://data.worldbank.org/.
